# Progress in Surgery

**Published:** 1900-02-03

**Authors:** 


### PROGRESS IN SURGERY.

### TUMOURS.

**Fibroma of the Abdominal Wall.**—The following account of these interesting tumours is an abstract from a paper by Dr. Jeff Miller.[1] The abdominal wall is seldom the site of tumours, and of those which occur fibromata are the most common. Of 70 cases of tumour of the abdominal wall collected by Sänger, 60 were fibromata. But they are rare tumours. Thus Leon Labbé observed only 10 cases in the Paris hospitals in 20 years. Guyon, in 1876, first recognised their true origin, viz., in aponeurotic structures. Labbé and Remy, in 1888, made the most extensive study of these tumours, collecting 100 cases, and most writers since have only confirmed their work. They occur almost exclusively in women, and are limited to the period of sexual activity, and intimately connected with childbirth. Thus, Senn states that, of 42 cases collected by Guerrein, 39 were in women. They usually appear soon after childbirth. Though any part of the abdominal wall may be affected, except the umbilicus or median line, yet usually they are found in the iliac regions or sheaths of the rectus muscle. In these situations the tissues are more liable to be injured during parturition. The tumours seem to start in the muscles or fascia. Histologically they resemble fibromata, being composed of young connective tissue cells with scanty intercellular substance. Removal is often followed by recurrence, especially if the enucleation has not been complete. They are usually encapsuled, but Senn states that the encapsulation is often incomplete. Sarcomatous degeneration occurs in some cases, therefore they should be removed early. The patients often first notice the tumours by accident as painless nodules. They may have a considerable bulk before being discovered, especially if the enlargement has been towards the abdominal cavity. If the growth has been towards the surface the tumour may be prominent and mobile under the skin. They become fixed upon muscular contraction. If deep-seated the diagnosis may be difficult, for the tumour may resemble pelvic tumours and cysts. Gummata and lipomata have to be differentiated If the tumour is near a bone the tendons and muscular fibres can be felt as tense and firm as a rope stretching between the growth and the skeleton. This constitutes the so-called bony pedicle of the early writers. They are distinguished from hydatid and urachal cysts by never being in the middle line nor at the umbilicus. They often grow slowly for some years, and then begin to grow quickly. When large, the peritoneum may be involved, necessitating opening of the peritoneal cavity for their removal, or even extensive resection of the peritoneum. Hernia is liable to follow the operation, and should be guarded against by suturing in tiers. If the peritoneum has been denuded over a large space it should be carefully brought into contact with the muscular layers with sutures, lest a cavity be left and sloughing follow. Dr. Miller gives notes of three cases. In the first case, that of a woman of 21, the tumour had been first noticed as a painless nodule two years previously. At the time of operation it was eight inches long and five broad, and situated in the right lumbar and inguinal regions. It was easily removed. The other cases were very similar.

**Angio-sarcoma.**—H. V. Würdemann,[2] assisted by Drs. Friend and Black, has written a paper on this class of tumour, and describes a case which has been very completely observed. The following remarks have been abstracted from this paper. Senn describes angio-sarcomata as usually more or less tuberous structures, with a consistence varying from that of a jelly-like mass to that of cartilage. On section, the surface presents an alveolar structure, and great vascularity, with occasional blood cysts and hæmorrhages. Microscopically the structure is reticular, seldom alveolar. The cells are arranged in strands corresponding to the blood vessels; they are epithelioid in shape, normally multinuclear. The ground substance is composed of connective tissue. The vessels are always capillary, and are numerous and large. Most frequently these tumours are found in the head and grow slowly, but recur rapidly. In five out of sixty cases the tumour gave rise to metastasis. Würdemann states that, despite favourable reports, it may be accepted as a truism that if cases of orbital sarcoma be watched long enough the neoplasm will be found to return, either in the original situation or elsewhere. If metastasis occurs it is usually the visceral organs that become affected. It seems likewise true that if the orbit be involved in general metastic sarcoma, this region is the original place involved, and the disease of other organs is caused by metastasis. Stirling shows that sarcoma affects both the uveal tract and the orbit in about equal proportions. The average age for uveal sarcoma is about 48, that of orbital cases about 28. Stirling concludes that it is probably less dangerous to have uveal sarcoma than sarcoma of the orbit. In the latter, if recurrence takes place the orbit is always affected, but this is not always the case in uveal sarcoma. The case related by the authors was one of multiple angio-sarcoma, with widespread metastasis and infiltration into various organs. The patient was a child of five and a half years of age, and first appeared complaining of a tumour of the left orbit which produced exophthalmos. The tumour was removed, the incision being through the upper eyelid. Two months later the eye protruded more than ever, and there was a swelling of the right parietal bone, and a tumour was found in the right hypogastrium. The eye was gradually pushed forward between the lids, and became inflamed and the cornea destroyed. She died five months after being first seen. An enormous mass of new growth was found in the abdomen matted to the liver, stomach, duodenum, and pancreas. Both the cranial tumours had destroyed the bone tissue, and were adherent to the dura mater. A portion of the brain anteriorly had been destroyed by the up-growth of the tumour from the roof of the orbit. It was remarkable that such an extensive involvement of brain tissue and membranes did not lead to conspicuous symptoms. The continuance of functional activity in the involved abdominal organs was also remarkable. The microscope showed the tumour to be an angio-sarcoma. A copious billiography is appended to this interesting paper.

**The Treatment of Inoperable Tumours.**—McFerly[3] has tried formalin in the treatment of an inoperable case of laryngeal epithelioma, with results that are interesting, though the growth was not cured. McFerly was encouraged to try this method, which he had previously had in mind for some years, by reading a published case of Dr. Mitchell's in the *British Medical Journal* of February, 1899. Mitchell's case was one of epithelioma of the cheek, which was a second recurrence after removal, and which two experienced surgeons had declined to operate upon. The growth was as large as a man's fist, and had forced its way through the integument at one part, from which constant bleeding occurred. To control this a small pad of absorbent cotton soaked in 20 per cent. solution of formaldehyde was applied to the raw surface, covered with gutta-percha and held in place by a bandage, the surrounding skin being protected by caoutchouc. The hæmorrhage was stopped, and in 24 hours hardening and necrosis had spread a quarter of an inch from the surface. This was scooped out, and the treatment daily repeated, leading in a short time to destruction of the growth, and finally permitting its complete removal without the loss of blood. McFeely's patient, a man of 53, had a tumour of the left side of the neck growing beneath the sterno-mastoid nearly opposite the thyroid cartilage, and another smaller one on the right side. There was marked aphonia from what proved to be an ulcerating epithelioma of the larynx. The tumour was injected at first with five minims of a 25 per cent. formalin solution. A few minutes' pain followed, though local anæsthesia had been previously induced. In succeeding injections the proportion of water was gradually diminished, until finally 25 minims of pure formalin without water were used. The latter injection seemed to give no more pain than the first. Although 60 minims of formalin were injected into a space of three square inches between March 6th and 9th there seemed not the least sign of hardening, but rather the tumour became daily softer. For some days before death which occurred on March 16th from asphyxia and collapse, there was trouble with the tracheotomy tube which the patient wore becoming blocked with fluid. Two centres of degeneration were found in the mass at the post-mortem. The skin over one had sloughed previously to his death, and watery discharge had escaped. The escape of similar watery discharge from degeneration of the tumour into the trachea had caused the fatal asphyxia. The following conclusions were stated: That up to half a drachm, of pure formalin can be injected without toxic symptoms resulting; that it is a powerful styptic, and does not seem liable to produce clotting or embolism; 'that used undiluted it is quite as safe as diluted, and seems to produce an anæsthetic effect more quickly; that it seems rather to retard cell growth in malignant tumours than to stimulate them to more rapid growth like some irritants.

Meriwether,[4] in some remarks upon Coley's treatment of inoperable tumours, says that he has collected 216 cases of sarcoma thus treated (148 were personal cases of Coley's), and that in them 26½ per cent. were cured and 43 per cent. benefited, and two or three cases of epithelioma and carcinoma appear to have been cured. No doubt these statistics are too favourable, for many unsuccessful cases will have remained unpublished. Two or three cases have died from pyogenic infection.

**Cyst of the Urachus.**—Ferguson[5] describes one more instance of this rare disease. The patient was a man of about 47. A swelling had been recently observed in the hypogastrium which was painful, and the man had frequent desire to micturate. The swelling reached two inches above the umbilicus. At the operation it was found to be under the deep fascia of the parietes. The contents were watery. The lower portion extended deeply into the pelvis, and could not be enucleated; but it was found that the whole of the inner wall, or secreting surface, could be peeled off, except near the umbilicus. The outer wall of the posterior part of the cyst was folded forward, and the sides stitched together at the level of the abdominal wound and excised. The lateral parts left were closed by running sutures, and the superficial layers of the parietes closed separately. A pocket was left at the umbilicus, where the secreting membrane could not be excised, and packed. Later this was allowed to become infected, so as to destroy the secreting surface. Three months after the operation there was a firm cicatrix and no urinary fistula.

[1] Med. Rec., June, 1899. [2] Amer. Jour. of Med. Sci., June, 1899. [3] Brit. Med. Jour., July, 1899. [4] Charlotte Med. Jour., June, 1899. [5] Brit. Med. Jour., June, 1899.

